# Moral Insanity

**Published:** 1857-04

**Authors:** J. Gotham

**Affiliations:** New York


### SELECTIONS.

From Medical & Surgical Reporter.

### "*Moral Insanity.*"

New York, January, 1857.

*Mr. Editor:* Moral insanity has, during the past month, been the great topic of discussion in and out of the profession, in this vicinity. It is talked of in the cars, it is discussed in the saloons, it is the staple on 'change, at the corners of the streets, in the halls of science, and in the courts of law. Since the last general election, nothing has so stirred the depths of society as this. The newspapers still ring with it; long leaders meet the eye, headed with those portentous words; the press groans under its influence; the shop windows are darkened with caricatures of it; tales of "fiction founded on fact," with this subject for the basis, fill long columns in the Sunday papers, and in staring capitals, a foot or more in length, it glares upon the walls and fences, in the form of advertisements, all because two legal gentlemen got their heads together to keep a notorious rascal out of State Prison, on the plea of "Moral Insanity." That was the talismanic word that was to break his shackles, and set him free to roam at large once more among the innocent money-changers of Wall Street.

A new form of mental disorder has become inaugurated into our text-books of insanity—*Monomania Huntingtoniensis*—in plain English, a propensity to write other people's names to promisory notes, and raise the money with them.

For about a year past an individual named Huntington has been preying with most consummate dexterity upon certain parties in Wall Street; raising by forged notes, money, to the extent, it is said, of about five millions of dollars, covering up his criminal acts, from time to time, by fresh forgeries of securities, and paying large percentages on the borrowed thousands. The money he spent sumptuously in riotous living, until, by a miscalculation of a month, as to the time when one of the alleged notes would fall due, the trouble burst, and that night our hero slept in the Tombs. So plain and abundant were the evidences of guilt, that no possible chance of escape remained to the criminal, but the desperate one which none but a lawyer of the boldest and most acute character would dare to raise, as a "plea in bar." Such astounding and long-continued criminalities could only have been conducted by a lunatic! To confess the whole, to magnify the crime, and then set up, before the jury, the plea of insanity, was their forlorn, their only hope. But what evidence of this, could they adduce? With a shrewdness more profound than the thought itself, they succeeded in inveigling into their plans two notable gentlemen of the medical profession, who, themselves above reproach or suspicion, were too easily led into the meshes prepared for them by these cunning gentlemen of the law. They visited the prisoner in his cell, conversed with him an hour or so, and although entirely unacquainted with him before, came from the interview prepared to give their professional opinions to the jury and to the world, that the prisoner was a *monomaniac*, whose insanity showed itself in an uncontrollable propensity, to use the brokers' phrase, "to make paper." Alas! for professional wisdom! Who is a criminal, if Huntington is insane? Answer, shade of Pinel! Answer, Esquirol and Miss Dix!

Of the medical gentlemen upon whose names and opinions the defence relied for an acquittal, one has been a tower of strength heretofore among us in Surgery, and the other known as a professor of Obstetrics. How high the first stood in public estimation, you may judge from the fact that when his evidence went forth on the wings of the press, the next day, not only all money-dom, but all prison-dom, and all kinds of *dom*, were thrown into a ferment of astonishment; some fearing lest a great criminal should escape, others hoping that crime of every sort might now be committed with impunity. Fortunately for the stability of society, the jury exercised their common sense, and the honest portion of the community breathed free again, when they returned their verdict of "guilty."

There is a moral in this case which should be treasured by all, especially by medical men: never give an opinion on a subject upon which you have little or no experience, especially when a cunning lawyer has a point to gain thereby. It is very plain to all that had the suspicion of the criminal's insanity rested upon any substantial basis, his counsel should, and doubtless would, have called upon the prosecuting attorney to unite in an investigation of the case. Instead of this, the whole idea was kept *sub rosa*, until the defence opened, and then it burst upon the community in surprise, especially when the two medical men took the stand. Happily the moral storm which such plea, if successful, would have raised, was averted.

It is due to these medical men to say, that, in spite of the public clamor, they still maintain, as honest men should, irrespective of consequences, their conviction of the unsoundness of mind of the prisoner, and a candid listener to their reasons cannot but see that they have some ground for the opinion they gave in court. It is alleged that on both the father's and mother's side there have been and are cases of confirmed lunacy in the family, which, taken in connection with his extravagantly wild and reckless course of life, and his stolid indifference to the consequences, form the basis of their conclusions. They had, as medical men, nothing whatever to do with the course pursued by the prisoner's counsel, nor were they called upon to shape their opinions in accordance with the wishes of either prosecuting counsel or witnesses, and they reason now, that if their opinions had been the ground of an actual acquital by the jury, blame could not attach to them, and society must provide for its own protection against further depredations of the same kind, but in what manner it is not for them to say. As medical men, it was their simple duty and privilege to give an opinion on the case, just as they would in any other. Whether that opinion is correct is a just matter of criticism, and there it must end.

### THE ACADEMY OF MEDICINE

Has done itself the honor of raising, for the second time, to the office of President, that veteran in surgery, Dr. Valentine Mott. Now past the allotted threescore and ten years, this unequalled master of the scalpel still bears as erect a figure, as healthful a countenance, a hand as steady, and an eye as strong as ever. He has lately tied the carotid artery for the forty-fourth time, in the living subject.

THE NEW YORK STATE MEDICAL SOCIETY

Will hold its first semi-centennial meeting in February, in Albany, when a *good time* is fully expected. I shall endeavor to be on hand, and give your readers some accounts of the deeds done in the body, as well as in the spirit. Respectfully,

J. GOTHAM, Jr., M. D.

